# Phosphoproteome Analysis Reveals Differential Mode of Action of Sorafenib in Wildtype and Mutated FLT3 Acute Myeloid Leukemia (AML) Cells*[Fn FN2]

**DOI:** 10.1074/mcp.M117.067462

**Published:** 2017-04-27

**Authors:** Catrin Roolf, Nikolaj Dybowski, Anett Sekora, Stefan Mueller, Gudrun Knuebel, Andreas Tebbe, Hugo Murua Escobar, Klaus Godl, Christian Junghanss, Christoph Schaab

**Affiliations:** From the ‡Department of Medicine, Clinic III-Hematology/Oncology/Palliative Care, Rostock University Medical Center, University of Rostock, 18057 Rostock, Germany;; §Evotec (München) GmbH, 82152 Martinsried, Germany;; ¶Department of Proteomics and Signal Transduction, Max-Planck Institute for Biochemistry, 82152 Martinsried, Germany

## Abstract

Constitutively activating internal tandem duplication (ITD) alterations of the receptor tyrosine kinase FLT3 (Fms-like tyrosine kinase 3) are common in acute myeloid leukemia (AML) and classifies FLT3 as an attractive therapeutic target. So far, applications of FLT3 small molecule inhibitors have been investigated primarily in FLT3-ITD^+^ patients. Only recently, a prolonged event-free survival has been observed in AML patients who were treated with the multikinase inhibitor sorafenib in addition to standard therapy. Here, we studied the sorafenib effect on proliferation in a panel of 13 FLT3-ITD^−^ and FLT3-ITD^+^ AML cell lines. Sorafenib IC50 values ranged from 0.001 to 5.6 μm, whereas FLT3-ITD^+^ cells (MOLM-13, MV4-11) were found to be more sensitive to sorafenib than FLT3-ITD^−^ cells. However, we identified two FLT3-ITD^−^ cell lines (MONO-MAC-1 and OCI-AML-2) which were also sorafenib sensitive. Phosphoproteome analyses revealed that the affected pathways differed in sorafenib sensitive FLT3-ITD^−^ and FLT3-ITD^+^ cells. In MV4-11 cells sorafenib suppressed mTOR signaling by direct inhibition of FLT3. In MONO-MAC-1 cells sorafenib inhibited the MEK/ERK pathway. These data suggest that the FLT3 status in AML patients might not be the only factor predicting response to treatment with sorafenib.

In acute myeloid leukemia (AML)^1^ the *Fms like tyrosine kinase 3* (FLT3) gene is frequently altered by the insertion of internal tandem duplications (ITD) in the juxtamembrane domain or by point mutations in the tyrosine kinase domain (TKD). These genetic alterations lead to an aberrant activation of downstream signaling proteins and promote cell proliferation of AML cells (1).

Deregulated kinases are promising targets in the treatment of cancer. Numerous FLT3 kinase inhibitors such as lestaurtinib (CEP-701) (2), midostaurin (PKC412) (3), and quizartinib (AC220) (4) have been developed and evaluated either in clinical trials as monotherapy or in combination with standard chemotherapeutic protocols in the last years.

Sorafenib is a multikinase inhibitor targeting different receptor tyrosine kinases including FLT3, vascular endothelial growth factor receptor (VEGFR), Kit and RET, which play an important role during myeloid cell differentiation (5). Several preclinical studies have demonstrated that AML cells with activating FLT3 receptor mutations are sensitive against sorafenib (6–8). Recently, sorafenib has been studied as monotherapy (9) or in combination with chemotherapeutics in clinical trials (10–12). Indeed, response rates for sorafenib in patients with FLT3-ITD^+^ are often higher than in patients without FLT3 alterations, but significant differences in overall survival have not been observed (10). Especially, elderly patients did not benefit from a sorafenib therapy (12). However, results of the randomized SORAML study showed a prolonged event-free survival in AML patients (< 60 years), who were treated with sorafenib in addition to standard induction and consolidation therapy (13). Of interest, only 17% of all patients in this study had the status of FLT3-ITD^+^. Therefore, FLT-3 ITD alone may not be sufficient to predict sorafenib response and the addition of other biomarkers may be required to improve the prediction accuracy. Here, we hypothesized, that the activation of other protein kinases beside FLT3 might predict the AML cell responsiveness to sorafenib.

Advances in sample processing, mass spectrometry, and computer algorithms have enabled the application of mass spectrometry-based proteomics to monitoring phosphorylation events on a global scale, allowing the identification and quantification of thousands of phosphorylation sites in a single experiment (14–17). When applied to cells treated with small molecules or antibodies, these methods allow the unbiased analysis of the mode of action of these agents (18–20). Recently, phosphoproteomics was also applied in the context of AML to study the mode of action of kinase inhibitors (21, 22) or to discover predictive biomarker candidates for kinase inhibitors (23). In the present study, we analyzed the effects of the multityrosine-kinase sorafenib on a panel of AML cell lines with different FLT3 status. Moreover, a phosphoproteome analysis was performed to understand the different modes of action in sorafenib sensitive and less sensitive AML cell lines. Our results show that subsets of both FLT3 wild-type and ITD mutated cell lines respond to treatment with sorafenib. However, the responses in these cell lines are mediated through different modes of action. Thus, beside the FLT3 status, the activation of certain signaling pathways might be used to predict the responsiveness of AML cells to treatment with sorafenib. Furthermore, we used a chemical proteomics approach to identify protein binding partners of sorafenib with differential binding affinity or differential expression in FLT3-ITD and FLT3 wild-type cell lines. Our data identified novel target pathways of sorafenib in AML cells.

## MATERIALS AND METHODS

### 

#### 

##### Cell Lines and Cell Culture Conditions

A panel of 13 AML cell lines with different morphology and FLT3 status (Table I) was used to study the effect of sorafenib treatment. All cell lines were purchased from Deutsche Sammlung von Mikroorganismen und Zellkultur (DSMZ, Braunschweig, Germany) and cultured according to the supplier's protocols. Stocks of cryopreserved cells were applied quickly after receipt from DSMZ and cells were passaged for less than 6 month. Cell lines were carefully checked for immune phenotype using flow cytometry and morphologic consistency by microscope. In addition, cell cultures were checked for mycoplasma contamination using Mycoalert Detection Kit (Lonza, Basel, Switzerland).

NB-4, HL-60, MOLM-13, MV4-11, NOMO-1, and KG-1 cell lines were cultured Roswell Park Memorial Institute (RPMI) 1640 medium (Biochrom AG, Berlin, Germany), supplemented with 10% fetal bovine serum (FBS) (PAA Laboratories GmbH, Pasching, Austria) and 100 μg/ml penicillin and streptomycin (P/S) (PAA Laboratories GmbH). MONO-MAC-1 cell line was cultured in RPMI 1640, supplemented with 10% FBS, 2 mm l-Glutamine (Biochrom AG), 1 mm Na-Pyruvate (Biochrom AG) and 100 μg/ml P/S. PL-21 and SKM-1 cell lines were cultured in RPMI 1640, supplemented with 20% FBS and100 μg/ml P/S. OCI-AML-2 cell line was cultured in alpha Minimum Essential Eagle's medium supplemented with 10% FBS and 100 μg/ml P/S. M-07e cells were cultured in RPMI 1640, supplemented with 20% FBS, 10 ng/ml GM-CSF (Bayer Healthcare Pharmaceuticals, LLC Seattle, WA) and and100 μg/m P/S. OCI-M1 and SH-2 cell lines were cultured in Iscove's Modified Dulbecco's Medium supplemented with 20% FBS and 100 μg/mg P/S. Cultures were maintained in a 5% CO_2_ humidified atmosphere at 37°C.

##### Screening for FLT3-ITD and FLT3-TKD Mutations

Genomic DNA was extracted from all cell lines using NucleoBond CB 100 (Macherey-Nagel, Dueren, Germany) and screened for FLT3-ITD and FLT3-TKD mutations. The presence of an ITD was confirmed by PCR amplification of the juxtamembrane domain from FLT3 exons 14 to 15 with a fluorescently labeled primer followed by fragment analysis using ABI Prism 3100 Avant Genetic Analyzer (Applied Biosystems, Darmstadt, Germany).

Point mutations at codon 835 and 836 were analyzed by amplification of FLT3 exon 20 followed by restriction fragment length polymorphism with EcoRV and gel electrophoretic analysis of cleavage products as previously reported (24).

##### Inhibitors

Sorafenib was purchased from LC Laboratories (Woburn, MA). Linifanib and vandetanib were obtained from Hycultec GmbH (Beutelsbach, Germany). All inhibitors were dissolved in dimethyl sulfoxide (DMSO). Stock solutions were prepared with a concentration of 10 mm and stored at −20 °C. Working solutions were prepared freshly by diluting stock solution with media.

##### Proliferation Studies

Cells (3.33 × 10^5^/ml) were seeded in 24-well or 96-well plates (Nunc, Langenselbold, Germany) and incubated with increasing concentrations of sorafenib, linifanib, or vandetanib (0.001–10 μm) for up to 72 h. Control cells were cultured in a medium containing the same concentration of DMSO as the treated cells. After treatment, cell counts were determined using trypan blue staining. Metabolic activity was determined using tetrazolium compound WST-1 (Roche Applied Science, Mannheim, Germany) as previously described (25). The IC50 value, at which 50% of the cell growth is inhibited compared with DMSO control, was calculated with probit analysis using the SPSS software, version 22 (IBM, Ehningen, Germany).

##### May-Grünwald Giemsa Staining

For examination of cell morphology, cells were fixed on glass slides in 200 μl cell suspension (based on DMSO treated cells) using a Shandon 3 Cytospin centrifuge (Shandon, Frankfurt/Main, Germany). After centrifugation, glass slides were air-dried and incubated with May Gruenwald and Giemsa solution (Merck, Darmstadt, Germany) as previously reported (26). Cell morphology was examined and visualized with Evos XL Core Imaging System (Life technologies, Darmstadt, Germany).

##### Analysis of Apoptosis

For analysis of apoptosis, cells were stained with Annexin V-Fluorescein isothiocyanate (FITC) (BD Biosciences, Heidelberg, Germany) and propidium iodide (PI) (Sigma Aldrich, St. Louis, MO) and analyzed by flow cytometry as previously described (25). Measurement of early apoptotic (Annexin V-FITC^+^ and PI^−^) and late apoptotic cells (Annexin V-FITC^+^ and PI^+^) was performed using FACSCalibur (Becton and Dickinson, Heidelberg, Germany).

##### Phosphoproteomics Analysis

For phosphoproteomics MV4-11, MONO-MAC-1, and SKM-1 cells were cultured in SILAC media containing 60.6 mg/ml Arg^0^ and 50 mg/ml Lys^0^ (light label), 62.3 mg/ml Arg^6^ and 61.1 mg/ml Lys^4^ (medium label), and 63.4 mg/ml Arg^10^ and 62.2 mg/ml Lys^8^ (heavy label). SILAC media were supplemented with 10% dialyzed fetal bovine serum (Invitrogen, Carlsbad, CA), 1% penicillin and streptomycin (Biochrom AG, Berlin, Germany), 1% glutamine (PAA, Pasching, Austria) and 1% sodium pyruvate (PAA). Cells were cultured for at least seven cell doublings to reach > 95% of incorporation of amino acids arginine and lysine, before treatment for 2 hours with DMSO, 0.01 or 0.2 μm sorafenib, respectively. Cells were lysed in ice-cold lysis buffer (8 M urea, 75 mm NaCl, 50 mm Tris pH 8.2, 5 mm EDTA, 5 mm EGTA, 10 mm sodium pyrophosphate, 10 mm NaF, 10 mm beta-glycerophosphate, 2 mm Na_3_VO_4,_ phosphatase inhibitor mixture 1 and 2 (Sigma-Aldrich) and protease inhibitor mixture tablet (Roche Applied Science, Mannheim, Germany)) and sonicated three times for 1 min on ice.

Lysates from the differently SILAC-encoded cells were pooled, reduced with dithiothreitol, alkylated with iodoacetamide and digested in-solution with endoproteinase Lys-C (Wako, Neuss, Germany) and trypsin (Promega, Mannheim, Germany) as described before (27). Lys-C and trypsin were used in excess to the protease inhibitors. Tryptic peptides were desalted using reversed-phase 500 mg C18 SepPak cartridges (Waters, Eschborn, Germany) and fractionated by SCX chromatography (250 × 9.4 mm polySULFOETHYL A column, 200 Å pore size, 5 μm particle size (PolyLC)) operated with ÄKTA explorer system (GE Healthcare, München, Germany) as described previously (28). The resulting twelve peptide samples were desalted using reversed-phase 100 mg C18 SepPak cartridges (Waters) before phosphopeptide enrichment with immobilized metal affinity chromatography (IMAC) in two successive enrichment steps essentially as described before (28–30). The resulting 24 samples per cell line were subjected to LC-MS/MS analysis. Phosphopeptide-enriched samples were loaded onto a reverse phase analytical column (packed in-house with C18 beads), resolved by an acetonitrile gradient using an Easy nLC system (Thermo Fisher Scientific, Waltham, MA) and directly electrosprayed via a nanoelectrospray ion source into an LTQ Orbitrap Velos mass spectrometer (Thermo Fisher Scientific) (27, 31). The Orbitrap mass spectrometer was operated in a data-dependent acquisition mode to automatically switch between full scans in the orbitrap mass analyzer (resolution *r* = 60.000) and the acquisition of CID fragmentation spectra (MS/MS mode) of the fifteen most abundant peptide ions in the linear ion trap (LTQ). For optimal phosphopeptide dissociation the multistage activation (MSA) modus was selected.

##### Bioinformatics Analysis

Identification and quantification of phosphorylation sites was performed using the MaxQuant software (version 1.4.0.6) (32) using the human Swissprot database including isoform sequences (version 07.2013 with 39,268 entries). Carbamidomethylation of cysteine was set as a fixed modification. Oxidation of methionine, N-terminal acetylation and phosphorylation on serine, threonine and tyrosine were set as variable modifications. The minimum required peptide length was seven amino acids, the minimum ratio count was set to two, the maximum number of missed cleavages was set to two, the main search tolerance was set to 4.5 ppm, and the MS/MS tolerance was set to 10 ppm. In-silico peptides were generated with “Trypsin/P,” which cleaves after lysine and arginine also if a proline follows. A false discovery rate (FDR) of 0.01 was selected for both protein and peptide identifications and a posterior error probability (PEP) below or equal to 0.1 for each peptide-to-spectral match was required. The match between runs option was enabled for a time window of 1 min. Phosphorylation sites were quantified using the SILAC ratios. In case multiple peptides were detected for the same site, the one with the least modifications was used for quantification.

The mass-spectrometry raw data and the MaxQuant output tables have been deposited to the ProteomeXchange Consortium (http://proteomecentral.proteomexchange.org) via the PRIDE partner repository with the data set identifier PXD004442.

Intensity ratios were transformed to reflect sorafenib *versus* DMSO control. Phosphorylation sites of low confidence (non-Class-I), reverse hits, or potential contaminants were removed. Significantly regulated phosphorylation sites were identified using the parametric MeanRank test (33). Significance testing was performed separately for all three cell models, using sites that were quantified in at least two of the three replicates. The MeanRank test intrinsically handles missing values and automatically corrects for multiple hypothesis testing. Phosphorylation sites were considered significantly regulated at an FDR of 0.05.

Enrichment analyses of gene ontology (GO) cellular component categories and KEGG pathways were performed for phosphoproteins regulated upon treatment with sorafenib compared with all phosphoproteins harboring quantified phosphorylation sites by non-conditional hypergeometric testing using Fisher's Exact Test. Enrichment of kinase substrates among significantly regulated phosphorylation sites was performed analogously. To correct for multiple hypothesis testing, FDR correction according to Benjamini-Hochberg was performed (FDR 0.05) (34).

##### Kinase Affinity Assay

Kinase selectivity of sorafenib in MV4-11 and MONO-MAC-1 cells was determined using KinAffinity as described (35, 36). Briefly, SILAC encoded cells were lysed in ice-cold buffer [20 mm HEPES pH 7.5; 150 mm NaCl, 0.25% Triton X-100, 1 mm EDTA, 1 mm EGTA, 1 mm DTT, 1 mm Na_3_VO_4,_ 10 mm NaF, protease inhibitors] and then incubated with KinAffinity beads. KinAffinity represents a set of different broad-selective kinase inhibitors immobilized to Sepharose beads, which serve as an affinity matrix for kinase enrichment. A concentration range (3 nm to 30 μm) of sorafenib was used to displace kinases from the matrix. Eluted proteins were separated by gel electrophoresis, digested by trypsin, and the resulting peptides were measured by mass spectrometry.

Identification and quantification of proteins was performed using MaxQuant software as described above. The data was further processed to remove nonspecific binders and to calculate dissociation constants k_D_ for the identified binding proteins.

##### Experimental Design and Statistical Rationale

The effect of sorafenib on cell growth, cell morphology, and apoptosis were initially tested in 13 AML cell lines with different morphology and FLT3 status. All experiments were conducted in triplicates and results within each experiment are described using mean ± standard deviation (S.D.). Significant effects (* α = 0.05) were determined by using the two-sample Student′s *t* test.

The phosphoproteomic profiling of MV4-11, MONO-MAC-1, and SKM-1 cells after treatment with DMSO, 0.01 or 0.2 μm sorafenib was also conducted in three biological replicates with label switching. Significantly regulated phosphorylation sites were identified using the parametric MeanRank test, which is a global, rank-based significance test intrinsically correcting for multiple hypothesis testing (33).

## RESULTS

### 

#### 

##### Sorafenib Sensitivity and FLT3 Status in AML Cells

Sensitivity to treatment with sorafenib was analyzed with increasing concentration (0.001–10 μm) in 13 AML cells with different French-American-British (FAB) classifications and FLT3 status (Table I). Treatment with sorafenib significantly inhibited the proliferation and decreased metabolic activity in FLT3-ITD as well as in FLT3 wild-type cells. The IC50 values of sorafenib ranged from 1 nm to 5.6 μm after 72 h treatment in the 13 AML cell lines and are summarized in Table I.

**Table I:** Characteristics of AML cell lines. The columns list the FAB classification, the FLT3-ITD and FLT3-TKD status and the cell growth IC50s

Cell lines	FAB	FLT3-ITD	FLT3-TKD	IC50 (72h)
MOLM-13	M5a	+	−	0.001 μm
MV4-11	M5a	+	−	0.001 μm
MONO-MAC-1	M5a	−	−	0.02 μm
OCI-AML-2	M4	−	−	0.08 μm
SH-2	M2	−	−	0.4 μm
KG-1	M6a	−	−	0.5 μm
NB-4	M3	−	−	1.1 μm
HL-60	M2	−	−	1.3 μm
OCI-M1	M6	−	−	1.7 μm
NOMO-1	M5a	−	−	1.9 μm
SKM-1	M5a	−	−	2.8 μm
PL-21	M3	+	−	4.1 μm
M-07e	M7	−	−	5.6 μm

In general, sorafenib was most potent in two AML cells with a constitutive FLT3 activation (MOLM-13 and MV4-11). Contrary, the PL-21 cell line has a FLT3-ITD mutation, but was less sensitive to sorafenib (IC50: 4.1 μm). Furthermore, we identified two cell lines (MONO-MAC-1 and OCI-AML-2) which were also sensitive to sorafenib and negative for FLT3-ITD or FLT3-TKD gene mutations. Thus, although there is a tendency that FLT3-ITD^+^ AML cells are more sensitive than FLT3 wild-type cells, we could identify exceptions to this general trend.

**Fig. 1.**
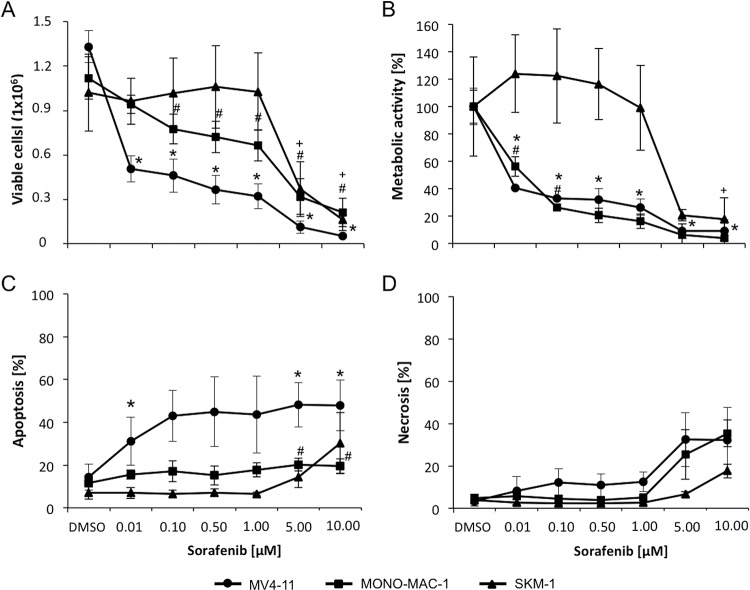
**Sensitivity to sorafenib is not depended on FLT3 status.** AML cell lines MV4-11, MONO-MAC-1 and SKM-1 were treated with 0.01–10 μm sorafenib for 72 h. MV4-11 (FLT3 ITD positive) and MONO-MAC-1 (FLT3 wild-type) are more sensitive to sorafenib than SKM-1 cells. The proliferation (*A*) and metabolic activity (*B*) was significantly inhibited on MV4-11 and MONO-MAC-1 cells from 0.1–10 μm sorafenib. Apoptotic (Annexin V-FITC^+^ and propidium iodide^−^) (*C*) and necrotic cells (Annexin V FITC^+^ and propidium iodide^+^) (*D*) were determined by flow cytometry. Highest amount of apoptosis rates were only observed in MV4-11 cells after treatment with sorafenib. Necrosis rates were increased with 5 and 10 μm sorafenib in MV4-11 and SKM-1. Results are displayed as the mean ± S.D. of three independent experiments. Symbols (*: MV4-11, #: MONO-MAC-1 and +: SKM-1) represents a statistically significance difference between sorafenib and DMSO treated cells with a *p* value <0.05.

We selected MV4-11, MONO-MAC-1, and SKM-1 for further studies as representatives of sensitive FLT3-ITD^+^, sensitive FLT3 wild-type, and insensitive FLT3 wild-type cell lines, respectively. A detailed analysis of these three cell lines in regards to cell count, metabolic activity, apoptosis, and necrosis rates after 72 h treatment is shown in Fig. 1. In both sorafenib sensitive cell lines, viable cells and metabolic activity decreased significantly in a dose dependent manner from 0.01 μm to 10 μm (MV4-11) and from 0.1 μm to 10 μm (MONO-MAC-1) with sorafenib in comparison to DMSO control cells. In SKM-1 cells metabolic activity declined significantly at the highest concentration of sorafenib (10 μm) only.

With lower concentrations of sorafenib (0.01–1.0 μm) apoptosis rates were elevated in MV4-11 (31.1 to 43.6%; DMSO: 14.4%) compared with MONO-MAC-1 (15.6 to 17.8%; DMSO: 11.6%). In MONO-MAC-1 cells a slight significant increase at apoptosis rates (19.5–20.3%) *versus* DMSO control cells (11.6%) could only be observed in cells treated with 5 and 10 μm sorafenib. Numbers of late apoptotic and necrotic cells increased with 5 μm sorafenib in MV4-11 (32.4%) and MONO-MAC-1 (25.3%) cell lines.

**Fig. 2.**
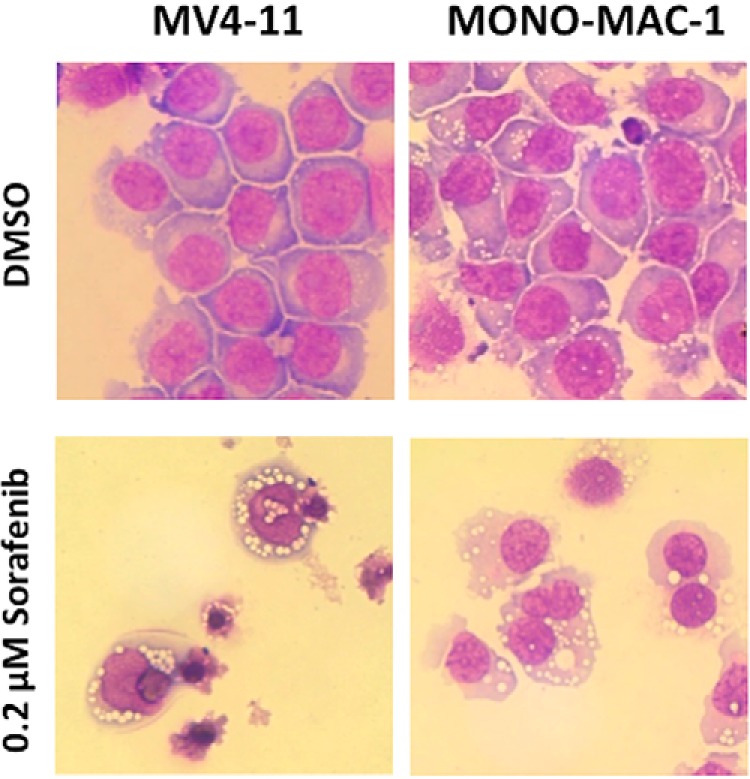
**Effects of sorafenib on cell morphology.** MV4-11 and MONO-MAC-1 cells were treated for up to 72 h with DMSO or sorafenib (0.2 μm) and stained using Pappenheim method to assess cellular morphology. Cytomorphology was altered in both cell lines. In MONO-MAC-1 cells an extensive cytoplasmic vacuolization could be observed whereas in MV4-11 cells, sorafenib induced more shrinkage characteristics with cell fragmentation into apoptotic bodies.

Alterations in cellular morphology of MV4-11 and MONO-MAC-1 cell lines after 72 h treatment with sorafenib are shown in Fig. 2. Response to sorafenib on MV4-11 cells appeared dissimilar and distinct from MONO-MAC-1 cells. After treatment, the FLT3-wild-type cell line MONO-MAC-1 exhibited plasma membrane blebbing and an extensive cytoplasmic vacuolization, whereas FLT3-mutated cells MV4-11 showed more shrinkage characteristics with cell fragmentation into apoptotic bodies.

##### Phosphoproteomics Analysis

**Fig. 3.**
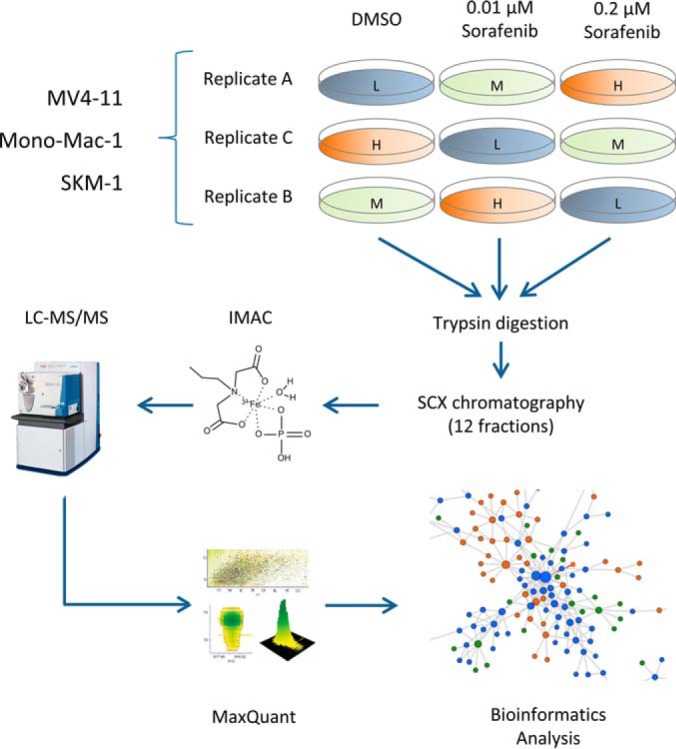
**Workflow for phosphoproteome analysis.** The AML cell lines MV4-11, MONO-MAC-1, and SKM-1 were SILAC-labeled, treated with DMSO, 0.01 μm sorafenib, and 0.2 μm sorafenib for two hours. Each experiment was performed in three replicates with a complete label switch according to the depicted experimental design. The pooled lysates of each experiment were subjected to a global, quantitative phosphoproteomics workflow (see Methods for more details). In total, 30,812 phosphorylation sites were identified in this study.

We were interested to see whether the phenotypic differences between sensitive FLT3 wild-type and FLT3-ITD cells is reflected by differences in regulation of signaling pathways in the respective cells. To this end, we utilized a global, un-biased, and quantitative phosphoproteome analysis based on mass-spectrometry and studied the effect of sorafenib treatment on the phosphoproteome in MV-11, MONO-MAC-1, and SKM-1 cells. The three cell lines were labeled by stable isotope labeling by amino acid in cell culture (SILAC) (37). Each cell line was grown in SILAC medium supplemented with light (Arg^0^/Lys^0^), medium (Arg^6^/Lys^4^), and heavy (Arg^10^/Lys^8^) forms of arginine and lysine until complete labeling (>95%) of all arginine and lysine residues was achieved. The cells were subsequently treated with DMSO, 0.01 μm sorafenib, and 0.2 μm sorafenib for 2 hours. Each experiment was repeated three times with a complete switch of the SILAC labels (Fig. 3). The pooled lysates of each experiment were subjected to a global, quantitative phosphoproteomics workflow using strong cation exchange chromatography and immobilized metal ion affinity chromatography followed by LC-MS/MS analysis.

In total, 30,812 phosphorylation sites were identified by MaxQuant (32) across all three profiled cell lines. 22,046 sites were rated as class I sites, *i.e.* sites that could be identified with high localization confidence. In average, 13,792 class I sites were quantified in at least 2 replicates per cell line (Table II). Only these sites were used for the following statistical analyses. The average localization probability was 0.957. The frequency distribution of the phosphorylated residues (serine 83.1%; threonine 15.3%; tyrosine 1.6%) was like the distributions observed in previous studies (38, 39).

**Table II:** Identified phosphorylation sites. The number of class-I sites detected in 1, 2, or 3 replicates per cell line. In total 22,046 class-I sites could be detected and 8378 sites were detected in at least 2 out of three replicates in each cell line

	3/3 Replicates	2/3 Replicates	1/3 Replicates	Total
MV4-11	11,544	2726	1980	16,250
Mono-Mac-1	9208	3948	3019	16,175
SKM1	9971	3791	2493	16,255

##### Bioinformatics Analysis

**Fig. 4.**
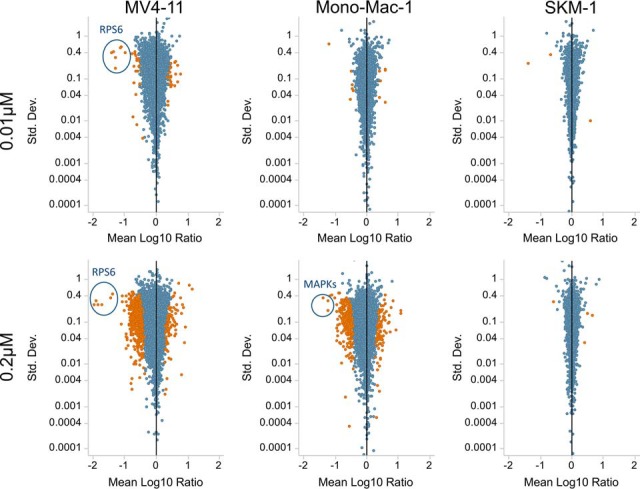
**Effect of sorafenib on the phosphoproteome.** The plots show the effect of treatment with 0.01 μm (top) and 0.2 μm (bottom) sorafenib on the phosphoproteome of MV4-11 (left), MONO-MAC-1 (middle), and SKM-1 (right). Each dot represents a phosphorylation site with its respective log-10 ratio between the sorafenib and DMSO treated cells and the standard deviation of the log-ratios across the three replicates. Significantly regulated phosphorylation sites are colored red (based on MeanRank test with 0.05 FDR).

The resulting SILAC ratios between sorafenib treated (0.01 and 0.2 μm) and DMSO treated cells were analyzed using the global, rank-based, one-sample, parametric MeanRank test (33). The effect of treatment with sorafenib on the phosphoproteome substantially differed between the three analyzed cell lines (Fig. 4). The FLT3 wild-type SKM-1 cell line was determined as almost insensitive to proliferation inhibition (IC50 = 2.8 μm), which was in accordance with the phosphoproteomics experiments that revealed almost no phospho-regulation upon sorafenib treatment. Only two phosphorylation sites (HNRNPLL pS61 and AKAP13 pS2561) were downregulated and three phosphorylation sites (TGFB1I1 pS403, SSFA2 pS593, FOXK1 pS253) were upregulated at the low concentration. Similarly, only eight phosphorylation sites were significantly regulated at the higher concentration. Interestingly, the five upregulated phosphorylation sites included pT185 and pY187 on ERK2, which control its kinase activity, pS222/pS226 on MEK1/MEK2, and pS245 and pS250 on the transcriptional repressor ETV3.

Within the FLT3 wild-type cell lines heterogeneous responses to sorafenib were observed. Whereas SKM-1 was not sensitive, MONO-MAC-1 cells showed growth inhibition in response to sorafenib at relatively low concentrations (IC50 = 0.02 μm). Nevertheless, only 18 phosphorylation sites significantly changed by treatment at the lower concentration (0.01 μm), whereas the phosphoproteome substantially altered at the higher concentration (0.2 μm) (Fig. 4). In total, 580 phosphorylation sites were significantly regulated at 0.2 μm (FDR 5%) with most sites being downregulated (∼ 78%). Among the sites with the strongest downregulation were pT181, pT185, and pY187 on ERK2 and pY204 on ERK1, which were downregulated by a factor of 15 to 25. Remarkably, the same phosphorylation sites were found to be upregulated in SKM-1 cells.

The FLT3-ITD positive cell line MV4-11 was sensitive to treatment with sorafenib at low concentrations (IC50 = 0.001 μm). The phosphoproteome analysis revealed substantial changes at both tested concentrations. 64 phosphorylation sites were significantly regulated at the low concentration, whereas the number further increased to 719 for the high concentration. Moreover, the degree of regulation strongly correlated between both concentrations (Pearson correlation 0.98, only sites significantly regulated at the low concentration were considered) and almost all phosphorylation sites that were significantly regulated at the lower concentration retain significance at the higher concentration. In contrast to the MONO-MAC-1 cell line, phosphorylations on ERK1/2 were not regulated in MV4-11. The strongest regulations were shown by six serine and one threonine phosphorylations on the 40S ribosomal protein S6 (RPS6) between amino acid position 235 and 247. These sites were downregulated 10–25-fold and 25–100-fold at the low concentration (0.01 μm) and high concentration (0.2 μm), respectively. RPS6 plays an important role in controlling cell growth (40) and has been shown to be activated in FLT3-mutated AML cells through activation of the mTOR signaling (41).

The database PhosphoSitePlus contains more than 8000 relationships between human kinases and substrates curated from literature (42). This comprehensive data set can be used to identify kinases that are located upstream of the regulated phosphorylation sites (43, 44). Indeed, a number of kinases, whose substrates were significantly enriched among the regulated phosphorylation sites, could be identified (Table III). This suggests that the activities of these kinases were inhibited by sorafenib treatment in the respective cell lines. Phosphorylations of the kinase MEK1 (MAP2K1) and its substrates ERK1/3 were found to be downregulated in FLT3 wild-type cell line MONO-MAC-1, which confirmed the finding that MEK-ERK signaling was inhibited by treatment with sorafenib in MONO-MAC-1. MEK1 could not be identified as regulated kinase in the FLT3-ITD cell line MV4-11. Instead, the ribosomal protein S6 kinases alpha-1, alpha-3, and beta-1 phosphorylating various sites on RPS6 and the serine/threonine-protein kinase AKT1 were identified in MV4-11. All four kinases play an important role the mTOR signaling pathway.

**Table III:** Upstream Kinases. Kinases, whose substrates are significantly enriched among regulated phosphorylation sites after treatment of the respective cell line with sorafenib. q-value: local FDR, regulated substrates: all known substrates (PhosphoSitePlus) of the respective kinases that are regulated

Cell Line	Kinase	q-Value	Regulated Substrates
MV4-11	AKT1	0.004	pBRCA1 (S694), pCHEK1 (S280), pDNMT1 (S143), pGSK3B (S9), pMETTL1 (S27), pPDCD4 (S457), pPFKFB3 (S461), pRANBP3 (S126), pTBC1D1 (T596), pZYX (S142)
	MAPK10	0.032	pMYC (T58,S62,S71)
	MAPKAPK2	0.018	pCDC25B (S353), pHSF1 (S121), pHSPB1 (S78), pPARN (S557), pRCSD1 (S179)
	RPS6KA1	2.2E-04	pEIF4b (S422), pGSK3B (S9), pRPS6 (S235,S236), pRPTOR (S719,S722), pRANBP3 (S126), pMETTL1 (S27)
	RPS6KA3	0.032	pGSK3B (S9), pRPS6 (S235,S236), pRANBP3 (S126)
	RPS6KB1	2.2E-05	pEIF4b (S422), pGSK3B (S9), pRPS6 (S235,S236,S240,S244), pNCBP1 (T21,S22)
MONO-MAC-1	MAP2K1	0.009	pMAPK1 (T185,Y187), pMAPK3 (T202,Y204)
	RPS6KA1	0.029	pYBX3 (S134), pRPS6 (S235,S236), pRPTOR (S719,S721,S722)
SKM-1	MAP2K1	0.004	pMAPK1 (T185,Y187)

**Fig. 5.**
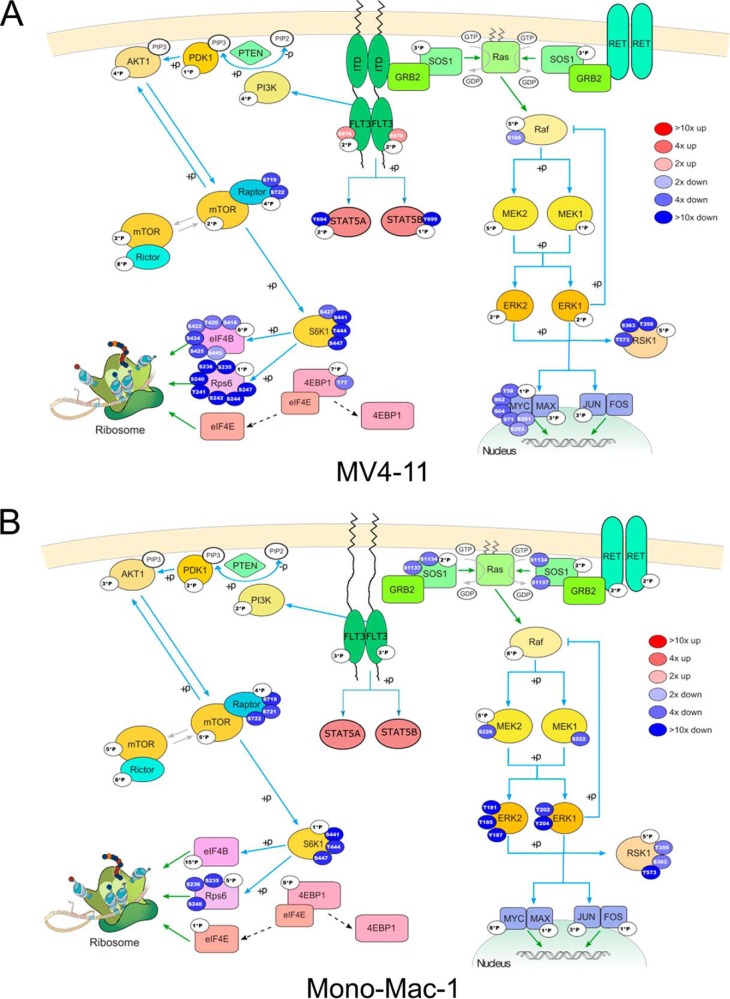
**mTOR and MEK/ERK pathways.** The significantly regulated phosphorylation sites for MV4-11 (*A*) and MONO-MAC-1 (*B*) treated with 0.2 μm sorafenib are mapped to the mTOR and MEK/ERK pathway downstream of the receptor tyrosine kinases FLT3 and RET. Each bubble represents a significantly regulated phosphosite; the ratio between DMSO and sorafenib treated cells is color coded (red: upregulated, blue: downregulated). White bubbles contain the number of detected but nonregulated sites (at 0.05 FDR).

This analysis suggested that the signaling pathways affected by sorafenib differs between the FLT3-ITD cell line MV4-11 on the one side and the wild-type cell lines SKM-1 and MONO-MAC-1 on the other side. Fig. 5 shows a detailed view on the mTOR and the MEK/ERK pathways in the two cell lines MV4-11 (Fig. 5*A*) and MONO-MAC-1 (Fig. 5*B*). Indeed, the MEK/ERK pathway was inhibited in MONO-MAC-1 but not in MV4-11, suggesting that sorafenib inhibited RAF or kinases upstream of RAF in MONO-MAC-1. Contrarily in MV4-11, mainly the mTOR pathway with RPS6, the S6 kinases and Raptor were strongly downregulated, whereas the regulation was much weaker in MONO-MAC-1 cells. This finding is in accordance with a downregulation of mTOR signaling through inhibition of FLT3 in MV4-11 cells and is supported by a more global comparison of the effect of sorafenib on a number of different cancer relevant pathways, transcription factors, and translation initiation factors (supplemental Figs. S1*A*, S1*B*, and S1*C*).

**Fig. 6.**
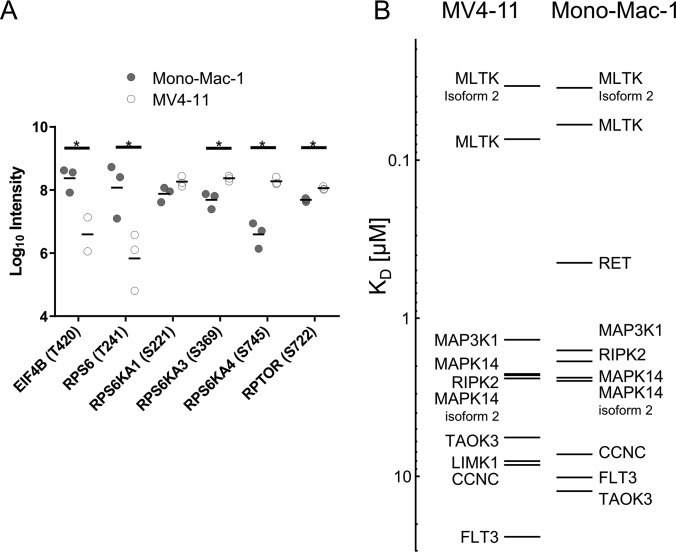
**Activation of mTOR pathway and sorafenib's kinase profile.** (*A*) Baseline phosphorylation (log 10) of proteins involved in the mTOR pathway. Each dot represents the log 10 intensity in each replicate DMSO experiment; the horizontal bars correspond to the respective mean. Lines with asterisks denote pairs for which the baseline phosphorylation is significantly different in MV4-11 and MONO-MAC-1 (*t* test, *p* < 0.05). (*B*) Kinase profile of sorafenib in MV4-11 and MONO-MAC-1. The horizontal bars represent the kinases identified as binders and their respective dissociation constants.

We see three possible explanations for the different effect of sorafenib on signaling events in MV4-11 cell line compared with SKM-1 or MONO-MAC-1. First, sorafenib targets different proteins; second, the baseline activity of the respective signaling pathways differs between these cell lines; or third a combination of both. To investigate the second possibility, we compared the baseline phosphorylation on proteins involved in the two pathways. Because the two cell lines were analyzed in separate SILAC experiments, we used the phosphorylation site intensities instead of SILAC-ratios to compare the baseline phosphorylation abundance between the two cell lines. Indeed, most of the proteins were differentially phosphorylated in the two cell lines (Fig. 6*A*). Whereas proteins located upstream in the pathway, such as Raptor and S6 kinases, were stronger phosphorylated in MV4-11, the proteins RPS6 and EIF4B showed the contrary. Although, the data seems to be not fully conclusive, it points to differences in the activation of the mTOR pathway in these two cell lines. One might speculate that the contrary activation of RPS6 and EIF4B may be caused by cross talks of other pathways.

##### Sorafenib Targets in FLT3-ITD^+^ and FLT3 Wild-type Cells

To analyze whether the observed effects in the analyzed cell lines might be explained by differential sorafenib targets, we applied chemical proteomics (35) to compare the binding affinity of sorafenib to proteins across the kinome between MV4-11 and MONO-MAC-1. To this end, SILAC-encoded cell extracts were used to enrich protein kinases by an affinity matrix consisting of a panel of broad-spectrum kinase inhibitor (KinAffinity beads). By performing competition experiments with sorafenib and subsequent quantitative mass spectrometry, the binding of more than 150 kinases could be quantified. Finally, using the Cheng-Prusoff equation the dissociation constants (*k_D_*-values) of sorafenib to each identified kinase was determined (see Methods for more details).

It is noteworthy, that the dissociation constants were determined in cell lysates in the physiological context of the corresponding cell line. These values may differ to dissociation constants determined *in vitro* with purified kinases because of various reasons. For example, the kinase may not be expressed in the investigated cell line or the compound-kinase interaction depends on the presence or absence of certain cofactors. Finally, the binding affinities determined for constructs expressing the kinase domain only may differ from the affinities to the full-length kinase. Davis *et al.* tested the interaction of 72 kinase inhibitors, including sorafenib, with a panel of 442 kinase domains *in vitro* (45). Whereas, they also determined MLTK (ZAK) and RET as two of the most affine sorafenib targets, other proteins, such as MAP3K1 or MAPK14 were either not included in the panel or showed a very low binding affinity. Other targets that showed a very high affinity *in vitro* (*e.g.* DDR1, DDR2) could not be detected in the KinAffinity approach; potentially because these proteins are not expressed in the investigated cell lines.

Fig. 6*B* shows a comparison of the dissociation constants of kinases in MV4-11 and MONO-MAC-1 cell lysates. In both cell lines MLTK (ZAK) had the highest affinity to sorafenib with k_D_ = 59 nm in MONO-MAC-1 and k_D_ = 74 nm in MV4-11. Other targets, which were identified in both cell lines with similar affinities, were MAPK14 (p38a), MAP3K1 (MEKK1), TAO3, RIPK2, and FLT3. Interestingly, the profiles revealed two differential targets. The tyrosine-protein kinase receptor RET was only identified in MONO-MAC-1 with a dissociation constant of k_D_ = 446 nm. RET is either not expressed or expressed at a level below the detection limit in MV4-11. Contrarily, LIMK1 was only detected in MV4-11 (k_D_ = 8 μm), but is too low expressed in MONO-MAC-1.

RET is known to activate several intracellular signaling cascades including the RAS/RAF pathway, which leads to activation of the MEK/ERK pathway (46) (see Fig. 5*B*). Thus, an inhibition of RET results in a suppression of the MEK/ERK pathway. Therefore, it is tempting to speculate that inhibition of RET activity by sorafenib is responsible for the observed downregulation of phosphorylations on MEK and ERK in MONO-MAC-1 cells.

**Fig. 7.**
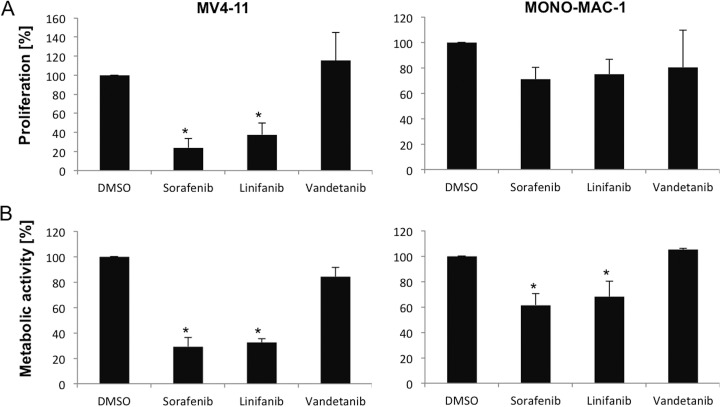
**Effects of linivanib and vandetanib on MV4-11 and MONO-MAC-1 cells.** MV4-11 and MONO-MAC-1 cells were treated for up to 72 h with DMSO, sorafenib (0.01 μm), linifanib (0.01 μm) or vandetanib (0.01 μm) to assess cell proliferation (*A*) and metabolic activity (*B*). In both cell lines a significant decrease of metabolic activity were induced with sorafenib and linivanib. Inhibition with RET kinase inhibitor vandetanib has no significant effects. Results are displayed as the mean ± S.D. of three independent experiments (* significant treatment effects *versus* DMSO treated cells).

In additional experiments, we investigated the effect of the more specific FLT3 and RET inhibitors vandetanib and linifanib in comparison with the multikinase inhibitor sorafenib on AML cells (45). To this end, MONO-MAC-1 and MV4-11 cells were treated with 0.01 μm vandetanib (RET inhibitor), 0.01 μm linifanib (FLT3 inhibitor) and 0.01 μm sorafenib for 72 h (see Fig. 7). Treatment with linifanib resulted in significant inhibition of proliferation and reduced metabolic activity with the most pronounced effects in MV4-11 cells. Here, metabolic activity decreased to 32% in linifanib and to 29% in sorafenib treated cells compared with DMSO treated cells (= 100%). In both cell lines, no significant effects on proliferation or metabolic activity were induced with RET inhibitor vandetanib.

## DISCUSSION

In the current study we investigated the effect of the multityrosine kinase inhibitor sorafenib on a panel of AML cell lines that differ in their FAB classification and their FLT3 mutational status. Although, cell lines with FLT3-ITD mutation that constitutively activates the FLT3 kinase tend to be more sensitive to treatment with sorafenib, also FLT3 wild-type cell lines, such as MONO-MAC-1 or OCI-AML-2, were sensitive in the proliferation assay with an IC50 below 100 nm. Furthermore, another FLT3-ITD^+^ cell line (PL-21) was resistant to treatment with sorafenib. Further analysis in apoptosis, metabolic, and necrosis assays revealed that the treatment with sorafenib leads to a substantially higher fraction of apoptotic cells in FLT3-ITD positive MV4-11 compared with MONO-MAC-1.

By performing a global, quantitative phosphoproteome analysis, the effect of sorafenib on activation or inhibition of signaling pathways was studied. Similar efforts to identify candidate biomarkers from phosphoproteomics analyses have recently been presented for dasatinib in nonsmall-cell lung cancer (39), for PI3K pathway inhibitors (47), and for the FLT3-inhibitor quizartinib in AML (23). Furthermore, the effect of FLT3-ITD mutation on compartment-dependent signaling in the FLT3-ITD 32D cell line was investigated (48). Here, we selected the in-sensitive, FLT3 wild-type cell line SKM-1, the sensitive FLT3 wild-type MONO-MAC-1, and the sensitive FLT3-ITD MV4-11 for this analysis. Not surprisingly, treatment with sorafenib had almost no effect on the phosphoproteome of the in-sensitive SKM-1 cells. Although many phosphorylations were significantly regulated in both sensitive cell lines, MV4-11 (719 phosphosites) and MONO-MAC-1 (580) at a concentration 0.2 μm, the affected pathways differ between the two cell lines. In the FLT3-ITD cell line MV4-11, proteins exhibiting the strongest downregulations are part of the mTOR signaling pathway. This finding is in agreement with a recent study showing that mTOR signaling is activated by FLT3 kinase and that mTOR signaling can be downregulated by inhibiting FLT3 (41, 49). In contrast, in the FLT3 wild-type cell line MONO-MAC-1, the pathway that is most strongly downregulated is the MEK/ERK signaling pathway.

We hypothesized that the different effect of sorafenib on signaling events in MV4-11 and MONO-MAC-1 can be explained by differing targets of sorafenib in these cell lines or by differing baseline activity of the respective signaling pathways. Indeed, we could show that the differential effect is caused by a combination of both. That is, the baseline activity of the mTOR signaling proteins Raptor and ribosomal protein S6 kinases is significantly lower in MONO-MAC-1 than in MV4-11. At the same time the comprehensive profiling of kinases for their binding affinity to sorafenib in these two cell lines, revealed that the tyrosine-protein kinase receptor RET is one of the high-affinity targets of sorafenib in MONO-MAC-1 (k_D_ = 446 nm), but is either not expressed or expressed below detection limit in MV4-11.

To confirm this hypothesis the effect of selective inhibition of RET but not FLT3 by vandetanib and of FLT3 but not RET by linifanib on cell growth of MV4-11 and MONO-MAC-1 was investigated. Although a solely specific RET inhibitor is currently not available, the comprehensive analysis of more than 70 known kinase inhibitors revealed vandetanib as the most specific RET inhibitor (45). Unfortunately vandetanib shows also high binding affinity to other kinases, such as EGFR, DDR1, MEK5 and ABL1. Application of vandetanib on MONO-MAC-1 showed not the expected inhibitory effect on cell proliferation. Because of the multi tyrosine kinase inhibitory activity of vandetanib an alternative feedback effect inducing pro-survival signals cannot be excluded. In perspective, inhibition of RET in MONO-MAC-1 with RNA interference or CRISPR/CAS9 could further validate the hypothesis. In contrast, FLT3 inhibition by linifanib on MV4-11 or MONO-MAC-1 cells showed comparable effects as with sorafenib exposure.

In summary, the current study showed that both, certain FLT3-ITD and FLT3 wild-type cell lines respond to treatment with sorafenib. Although the sensitivity trends to be lower in wild-type cells. The response in two representatives of both subclasses is mediated by different modes of action. Whereas sorafenib suppresses mTOR signaling most likely by directly inhibiting FLT3 in the FLT3-ITD cell line MV4-11, it suppresses MEK/ERK signaling in the FLT3 wild-type, but sensitive cell line MONO-MAC-1. Results of kinase inhibitor profiling using a chemical proteomics approach, indicate that blocking RET tyrosine-receptor kinase may be the cause of the inhibition of the MEK/ERK pathway. Our results suggest that the FLT3 status in AML patients might not be the only key determinant for the treatment of patients with sorafenib. Although, these novel results must be confirmed *in vivo* or in patient derived models in future studies, they are useful for considering potential combinatorial agents and for selecting candidates for biomarkers predicting response to treatment with sorafenib.

## DATA AVAILABILITY

The mass-spectrometry raw data and the MaxQuant output tables have been deposited to the ProteomeXchange Consortium (http://proteomecentral.proteomexchange.org) via the PRIDE partner repository with the data set identifier PXD004442.

## Supplementary Material

Supplemental Data

## Footnotes

^1^ The abbreviations used are:

AML: acute myeloid leukemia
FLT3: Fms-like tyrosine kinase 3
ITD: internal tandem duplications
MSA: multistage activation
PEP: posterior error probability.

## References

[1] Meshinchi S., and Appelbaum F. R. (2009) Structural and functional alterations of FLT3 in acute myeloid leukemia. Clin. Cancer Res. 15, 4263–4269 19549778 10.1158/1078-0432.CCR-08-1123 PMC2716016

[2] Levis M., Ravandi F., Wang E. S., Baer M. R., Perl A., Coutre S., Erba H., Stuart R. K., Baccarani M., Cripe l. D., Tallman M. S., Meloni G., Godley L. A., Langston A. A., Amadori S., Lewis I. D., Nagler A., Stone R., Yee K., Advani A., Douer D., Wiktor-Jedrzejczak W., Juliusson G., Litzow M. R., Petersdorf S., Sanz M., Kantarjian H. M., Sato T., Tremmel L., Bensen-Kennedy D. M., Small D., and Smith B. D. (2011) Results from a randomized trial of salvage chemotherapy followed by lestaurtinib for patients with FLT3 mutant AML in first relapse. Blood 117, 3294–3301 21270442 10.1182/blood-2010-08-301796 PMC3069671

[3] Fischer T., Stone R. M., Deangelo D. J., Galinsky I., Estey E., Lanza C., Fox E., Ehninger G., Feldman E. J., Schiller G. J., Klimek V. M., Nimer S. D., Gilliland D. G., Dutreix C., Huntsman-Labed A., Virkus J., and Giles F. J. (2010) Phase IIB trial of oral Midostaurin (PKC412), the FMS-like tyrosine kinase 3 receptor (FLT3) and multi-targeted kinase inhibitor, in patients with acute myeloid leukemia and high-risk myelodysplastic syndrome with either wild-type or mutated FLT3. J Clin Oncol. 28, 4339–4345 20733134 10.1200/JCO.2010.28.9678 PMC4135183

[4] Cortes J. E., Kantarjian H., Foran J. M., Ghirdaladze D., Zodelava M., Borthakur G., Gammon G., Trone D., Armstrong R. C., James J., and Levis M. (2013) Phase I study of quizartinib administered daily to patients with relapsed or refractory acute myeloid leukemia irrespective of FMS-like tyrosine kinase 3-internal tandem duplication status. J Clin Oncol. 31, 3681–3687 24002496 10.1200/JCO.2013.48.8783 PMC3804291

[5] Wilhelm S., Carter C., Lynch M., Lowinger T., Dumas J., Smith R. A., Schwartz B., Simantov R., and Kelley S. (2006) Discovery and development of sorafenib: a multikinase inhibitor for treating cancer. Nat. Rev. Drug Discov. 5, 835–844 17016424 10.1038/nrd2130

[6] Auclair D., Miller D., Yatsula V., Pickett W., Carter C., Chang Y., Zhang X., Wilkie D., Burd A., Shi H., Rocks S., Gedrich R., Abriola L., Vasavada H., Lynch M., Dumas J., Trail P., and Wilhelm S. M. (2007) Antitumor activity of sorafenib in FLT3-driven leukemic cells. Leukemia 21, 439–445 17205056 10.1038/sj.leu.2404508

[7] Zhang W., Konopleva M,, Shi Y., Mcqueen T., Harris D., Ling X., Estrov Z., Quintás-cardama A., Small D., Cortes J., and Andreeff M. (2008) Mutant FLT3: A direct target of sorafenib in acute myelogenous leukemia. J. Nat. Cancer Inst. 6, 184–198 10.1093/jnci/djm328 18230792

[8] Pratz K. W., Sato T., Murphy K. M., Stine A., Rajkhowa T., and Levis M. (2010) FLT3-mutant allelic burden and clinical status are predictive of response to FLT3 inhibitors in AML. Blood 115, 1425–1432 20007803 10.1182/blood-2009-09-242859 PMC2826764

[9] Borthakur G., Kantarjian H., Ravandi F., Zhang W., Konopleva M., Wright J. J., Faderl S., Verstovsek S., Mathews S., Andreeff M., and Cortes J. E. (2011) Phase I study of sorafenib in patients with refractory or relapsed acute leukemias. Haematologica 96, 62–68 20952518 10.3324/haematol.2010.030452 PMC3012766

[10] Ravandi F., Arana Yi C., Cortes J. E., Levis M., Faderl S., Garcia-Manero G., Jabbour E., Konopleva M., O'Brien S., Estrov Z., Borthakur G., Thomas D., Pierce S., Brandt M., Pratz K., Luthra R., Andreeff M., and Kantarjian H. (2014) Final report of phase II study of sorafenib, cytarabine and idarubicin for initial therapy in younger patients with acute myeloid leukemia. Leukemia 28, 1543–1545 24487412 10.1038/leu.2014.54 PMC4091714

[11] Macdonald D. A., Assouline S. E., Brandwein J., Kamel-Reid S., Eisenhauer E. A., Couban S., Caplan S., Foo A., Walsh W., Leber B. (2013) A phase I/II study of sorafenib in combination with low dose cytarabine in elderly patients with acute myeloid leukemia or high-risk myelodysplastic syndrome from the National Cancer Institute of Canada Clinical Trials Group: trial IND.186. Leuk. Lymphoma 54, 760–766 23061485 10.3109/10428194.2012.737917

[12] Serve H., Krug U., Wagner R., Sauerland M. C., Heinecke A., Brunnberg U., Schaich M., Ottmann O., Duyster J., Wandt H., Fischer T., Giagounidis A., Neubauer A., Reichle A., Aulitzky W., Noppeney R., Blau I., Kunzmann V., Stuhlmann R. Krämer A., Kreuzer K.-A., Brandts C., Steffen B., Thiede C., Müller-Tidow C., Ehninger G., and Berdel W. E. (2013) Sorafenib in combination with intensive chemotherapy in elderly patients with acute myeloid leukemia: results from a randomized, placebo-controlled trial. J. Clin. Oncol. 31, 3110–3118 23897964 10.1200/JCO.2012.46.4990

[13] Röllig C., Serve H., Hüttmann A., Noppeney R., Müller-Tidow C., Krug U., Baldus C. D., Brandts C. H., Kunzmann V., Einsele H., Krämer A., Schäfer-Eckart K., Neubauer A., Burchert A., Giagounidis A., Krause S. W., Mackensen A., Aulitzky W., Herbst R., Hänel M., Kiani A., Frickhofen N., Kullmer J., Kaiser U., Link H., Geer T., Reichle A., Junghanß C., Repp R., Heits F., Dürk H., Hase J., Klut I.-M., Illmer T., Bornhäuser M., Schaich M., Parmentier S., Görner M., Thiede C., von Bonin M., Schetelig J., Kramer M., Berdel W. E., and Ehninger G. (2015) Addition of sorafenib versus placebo to standard therapy in patients aged 60 years or younger with newly diagnosed acute myeloid leukaemia (SORAML): a multicentre, phase 2, randomised controlled trial. Lancet Oncol. 16, 1691–1699 26549589 10.1016/S1470-2045(15)00362-9

[14] Olsen J. V., Blagoev B., Gnad F., Macek B., Kumar C., Mortensen P., et al (2006) Global, in vivo, and site-specific phosphorylation dynamics in signaling networks. Cell 127, 635–648 17081983 10.1016/j.cell.2006.09.026

[15] Schaab C. (2011) Analysis of phosphoproteomics data. Methods Mol. Biol. 696, 41–57 21063940 10.1007/978-1-60761-987-1_3

[16] Sharma K., D'Souza R. C. J., Tyanova S., Schaab C., Wiœniewski J. R., Cox J., and Mann M. (2014) Ultradeep human phosphoproteome reveals a distinct regulatory nature of tyr and ser/thr-based signaling. Cell Rep. 8, 1583–1594 25159151 10.1016/j.celrep.2014.07.036

[17] Riley N. M., and Coon J. J. (2016) Phosphoproteomics in the age of rapid and deep proteome profiling. Anal. Chem. 88, 74–94 26539879 10.1021/acs.analchem.5b04123 PMC4790442

[18] Conradt L., Godl K., Schaab C., Tebbe A., Eser S., Diersch S., Michalski C. W., Kleeff J., Schnieke A., Schmid R. M., Saur D., and Schneider G. (2011) Disclosure of erlotinib as a multikinase inhibitor in pancreatic ductal adenocarcinoma. Neoplasia 13, 1026–1034 22131878 10.1593/neo.111016 PMC3223607

[19] Weigand S., Herting F., Maisel D., Nopora A., Voss E., Schaab C., Klammer M., and Tebbe A. (2012) Global quantitative phosphoproteome analysis of human tumor xenografts treated with a CD44 antagonist. Cancer Res. 72, 4329–4339 22777824 10.1158/0008-5472.CAN-12-0136

[20] Pan C., Olsen J. V., Daub H., and Mann M. (2009) Global effects of kinase inhibitors on signaling networks revealed by quantitative phosphoproteomics. Mol. Cell. Proteomics 8, 2796–2808 19651622 10.1074/mcp.M900285-MCP200 PMC2816010

[21] Alcolea M. P., Casado P., Rodriguez-Prados J. C., Vanhaesebroeck B., Cutillas P. R. (2014) Phosphoproteomic analysis of leukemia cells under basal and drug-treated conditions identifies markers of kinase pathway activation and mechanisms of resistance. Mol. Cell. Proteomics 11, 453–466 10.1074/mcp.M112.017483 PMC3412974 22547687

[22] Bertacchini J., Guida M., Accordi B., Mediani L., Martelli A. M., Barozzi P., Petricoin E., Liotta L., Milani G., Giordan M., Luppi M., Forghieri F., De Pol A., Cocco L., Basso G., and Marmiroli S. (2014) Feedbacks and adaptive capabilities of the PI3K/Akt/mTOR axis in Acute Myeloid Leukemia revealed by pathway selective inhibition and phosphoproteome analysis. Leukemia 28, 2197–2205 24699302 10.1038/leu.2014.123

[23] Schaab C., Oppermann F. S., Klammer M., Pfeifer H., Tebbe A., Oellerich T., Krauter J., Levis M., Perl A. E., Daub H., Steffen B., Godl K., and Serve H. (2014) Global phosphoproteome analysis of human bone marrow reveals predictive phosphorylation markers for the treatment of acute myeloid leukemia with quizartinib. Leukemia 28, 716–719 24247654 10.1038/leu.2013.347 PMC3948157

[24] Kiyoi H., Naoe T., Yokota S., Nakao M., Minami S., Kuriyama K., Takeshita A., Saito K., Hasegawa S., Shimodaira S., Tamura J., Shimazaki C., Matsue K., Kobayashi H., Arima N., Suzuki R., Morishita H., Saito H., Ueda R., and Ohno R. (1997) Internal tandem duplication of FLT3 associated with leukocytosis in acute promyelocytic leukemia. 11, 1447–1452 10.1038/sj.leu.2400756 9305596

[25] Schult C., Dahlhaus M., Ruck S., Sawitzky M., Amoroso F., Lange S., Etro D., Glass A., Fuellen G., Boldt S., Wolkenhauer O., Neri L. M., Freund M., and Junghanss C. (2014) The multikinase inhibitor Sorafenib displays significant antiproliferative effects and induces apoptosis via caspase 3, 7 and PARP in B- and T-lymphoblastic cells. BMC Cancer 10, 560 10.1186/1471-2407-10-560 PMC2972283 20950443

[26] Kretzschmar C., Roolf C., Langhammer T.-S., Sekora A., Pews-Davtyan A., Beller M., Frech M. J., Eisenlöffel C., Rolfs A., and Junghanss C. (2014) The novel arylindolylmaleimide PDA-66 displays pronounced antiproliferative effects in acute lymphoblastic leukemia cells. BMC Cancer. 14, 71 24502201 10.1186/1471-2407-14-71 PMC3922486

[27] Daub H., Olsen J. V., Bairlein M., Gnad F., Oppermann F. S., Körner R., Greff Z., Kéri G., Stemmann O., and Mann M. (2008) Kinase-selective enrichment enables quantitative phosphoproteomics of the kinome across the cell cycle. Mol. Cell. 31, 438–448 18691976 10.1016/j.molcel.2008.07.007

[28] Villén J., and Gygi S. P. (2008) The SCX/IMAC enrichment approach for global phosphorylation analysis by mass spectrometry. Nat. Protoc. 3, 1630–1638 18833199 10.1038/nprot.2008.150 PMC2728452

[29] Rappsilber J., Mann M., and Ishihama Y. (2007) Protocol for micro purification, enrichment, pre fractionation and storage of peptides for proteomics using StageTips. Nat. Protoc. 2, 1896–1906 17703201 10.1038/nprot.2007.261

[30] Dephoure N., Zhou C., Villén J., Beausoleil S. A., Bakalarski C. E., Elledge S. J., and Gygi S. P. (2008) A quantitative atlas of mitotic phosphorylation. Proc. Natl. Acad. Sci. U.S.A. 105, 10762–10767 18669648 10.1073/pnas.0805139105 PMC2504835

[31] Olsen J. V., Schwartz J. C., Griep-Raming J., Nielsen M. L., Damoc E., Denisov E., Lange O,. Remes P., Taylor D., Splendore M., Wouters E. R., Senko M., Makarov A., Mann M., and Horning S. (2009) A dual pressure linear ion trap Orbitrap instrument with very high sequencing speed. Mol. Cell. Proteomics 8, 2759–2769 19828875 10.1074/mcp.M900375-MCP200 PMC2816009

[32] Cox J., and Mann M. (2008) MaxQuant enables high peptide identification rates, individualized p.p.b.-range mass accuracies and proteome-wide protein quantification. Nat Biotechnol. 26, 1367–1372 19029910 10.1038/nbt.1511

[33] Klammer M., Dybowski J. N., Hoffmann D., and Schaab C. (2014) Identification of significant features by the global mean rank test. PLoS ONE 9, e104504 25119995 10.1371/journal.pone.0104504 PMC4132091

[34] Benjamini Y., and Hochberg Y. (1995) Controlling the false discovery rate: a practical and powerful approach to multiple testing. J. R. Stat. Soc. 57, 289–300

[35] Sharma K., Weber C., Bairlein M., Greff Z., Kéri G., Cox J., Olsen J. V., and Daub H. (2014) Proteomics strategy for quantitative protein interaction profiling in cell extracts. Nat. Methods 6, 741–744 10.1038/nmeth.1373 19749761

[36] Delehouzé C., Godl K., Loaëc N., Bruyère C., Desban N., Oumata N., Galons H., Roumeliotis T. I., Giannopoulou E. G., Grenet J., Twitchell D., Lahti J., Mouchet N., Galibert M.-D., Garbis S. D., and Meijer L. (2014) CDK/CK1 inhibitors roscovitine and CR8 downregulate amplified MYCN in neuroblastoma cells. Oncogene 33, 5675–5687 24317512 10.1038/onc.2013.513 PMC4087096

[37] Ong S. E., Blagoev B., Kratchmarova I., Kristensen D. B., Steen H., Pandey A., and Mann M. (2002) Stable isotope labeling by amino acids in cell culture, SILAC, as a simple and accurate approach to expression proteomics. Mol. Cell. Proteomics 1, 376–386 12118079 10.1074/mcp.m200025-mcp200

[38] Lundby A., Andersen M. N., Steffensen A. B., Horn H., Kelstrup C. D., Francavilla C., Jensen L. J., Schmitt N., Thomsen M. B., and Olsen J. V. (2014) In vivo phosphoproteomics analysis reveals the cardiac targets of β-adrenergic receptor signaling. Sci. Signal. 6, rs11 10.1126/scisignal.2003506 23737553

[39] Klammer M., Kaminski M., Zedler A., Oppermann F., Blencke S., Marx S., Muller S., Tebbe A., Godl K., and Schaab C. (2012) Phosphosignature predicts dasatinib response in non-small cell lung cancer. Mol. Cell. Proteomics 11, 651–668 22617229 10.1074/mcp.M111.016410 PMC3434785

[40] Chen B., Zhang W., Gao J., Chen H., Jiang L., Liu D., Cao Y., Zhao S., Qiu Z., Zeng J., Zhang S., and Li W. (2014) Downregulation of ribosomal protein S6 inhibits the growth of non-small cell lung cancer by inducing cell cycle arrest, rather than apoptosis. Cancer Lett. 354, 378–389 25199762 10.1016/j.canlet.2014.08.045

[41] Chen W., Drakos E., Grammatikakis I., Schlette E.J., Li J., Leventaki V., Staikou-Drakopoulou E., Patsouris E., Panayiotidis P., Medeiros L. J., and Rassidakis G. Z. (2010) mTOR signaling is activated by FLT3 kinase and promotes survival of FLT3-mutated acute myeloid leukemia cells. Mol. Cancer 9, 292 21067588 10.1186/1476-4598-9-292 PMC2993677

[42] Hornbeck P. V., Kornhauser J. M., Tkachev S., Zhang B., Skrzypek E., Murray B., Latham V., and Sullivan M. (2012) PhosphoSitePlus: a comprehensive resource for investigating the structure and function of experimentally determined post-translational modifications in man and mouse. Nucleic Acids Res. 40, D261–D270 22135298 10.1093/nar/gkr1122 PMC3245126

[43] Casado P., Rodriguez-Prados J. C., Cosulich S. C., Guichard S., Vanhaesebroeck B., Joel S., and Cutillas P. R. (2013) Kinase-substrate enrichment analysis provides insights into the heterogeneity of signaling pathway activation in leukemia cells. Sci. Signal. 6, rs6 23532336 10.1126/scisignal.2003573

[44] Domanova W., Krycer J., Chaudhuri R., Yang P., Vafaee F., Fazakerley D., Humphrey S., James D., and Kuncic Z. Unraveling kinase activation dynamics using kinase-substrate relationships from temporal Large-Scale phosphoproteomics studies. PLoS ONE 11, e0157763 10.1371/journal.pone.0157763 PMC4918924 27336693

[45] Davis M. I., Hunt J. P., Herrgard S., Ciceri P., Wodicka L. M., Pallares G., Hocker M., Treiber D. K., and Zarrinkar P. P. (2011) Comprehensive analysis of kinase inhibitor selectivity. Nat. Biotechnol. 29, 1046–1051 22037378 10.1038/nbt.1990

[46] Arighi E., Borrello M.G., Sariola H. (2005) RET tyrosine kinase signaling in development and cancer. Cytokine Growth Factor Rev. 16, 441–67 15982921 10.1016/j.cytogfr.2005.05.010

[47] Andersen J., Sathyanarayanan S., Di Bacco A., Chi A., Zhang T., Chen A., Dolinski B., Kraus M., Roberts B., Arthur W., Klinghoffer R. A., Gargano D., Li L., Feldman I., Lynch B., Rush J., Hendrickson R. C., Blume-Jensen P., and Paweletz C. P. (2010) Pathway-based identification of biomarkers for targeted therapeutics: personalized oncology with PI3K pathway inhibitors. Sci. Transl. Med. 2, 43ra55 10.1126/scitranslmed.3001065 20686178

[48] Choudhary C., Olsen J. V., Brandts C., Cox J., Reddy P. N. G., Böhmer F. D., Gerke V., Schmidt-Arras D.-E., Berdel W. E., Müller-Tidow C., Mann M., and Serve H. (2009) Mislocalized activation of oncogenic RTKs switches downstream signaling outcomes. Mol. Cell. 36, 326–339 19854140 10.1016/j.molcel.2009.09.019

[49] Perl A. E., Kasner M. T., Shank D., Luger S. M., and Carroll M. (2014) Single-cell pharmacodynamic monitoring of S6 ribosomal protein phosphorylation in AML Blasts during a clinical trial combining the mTOR inhibitor sirolimus and intensive chemotherapy. Clin. Cancer Res. 18, 1716–1725 10.1158/1078-0432.CCR-11-2346 PMC3306511 22167413

