# Supplementary material for: Phosphoproteome Analysis Reveals Differential Mode of Action of Sorafenib in Wildtype and Mutated FLT3 Acute Myeloid Leukemia (AML) Cells
Source: Mol Cell Proteomics. 2017 Apr 27;16(7):1365–76. doi: 10.1074/mcp.M117.067462 (PMC5500767; doi:10.1074/mcp.M117.067462)
Supplement: Supplemental Data [file supp_16_7_1365__index.html]

Phosphoproteome Analysis Reveals Differential Mode of Action of Sorafenib in Wildtype and Mutated FLT3 AML Cells — Phosphoproteome Analysis Reveals Differential Mode of Action of Sorafenib in Wildtype and Mutated FLT3 Acute Myeloid Leukemia (AML) Cells — Phosphoproteomics on Sorafenib Treated AML Cells — Supplemental Data 

# Phosphoproteome Analysis Reveals Differential Mode of Action of Sorafenib in Wildtype and Mutated FLT3 Acute Myeloid Leukemia (AML) Cells

## Supplemental Data

- Suppl. Fig. 1a (.pdf, 752 KB) - Cancer relevant pathways
- Suppl. Fig. 1b (.pdf, 489 KB) - Transcription factors
- Suppl. Fig. 1c (.pdf, 44 KB) - Translation initiation factors
- Suppl. Table 1 (.xlsx, 14.0 MB) - Phosphorylations in MV4-11, MONO-MAC-1, and SKM-1 in response to sorafenib treatment
