# Supplementary material for: Phosphoproteome Analysis Reveals Differential Mode of Action of Sorafenib in Wildtype and Mutated FLT3 Acute Myeloid Leukemia (AML) Cells
Source: Mol Cell Proteomics. 2017 Apr 27;16(7):1365–76. doi: 10.1074/mcp.M117.067462 (PMC5500767; doi:10.1074/mcp.M117.067462)
Supplement: Supplemental Data [file 10.1074_M117.067462_mcp.M117.067462-1.pdf]

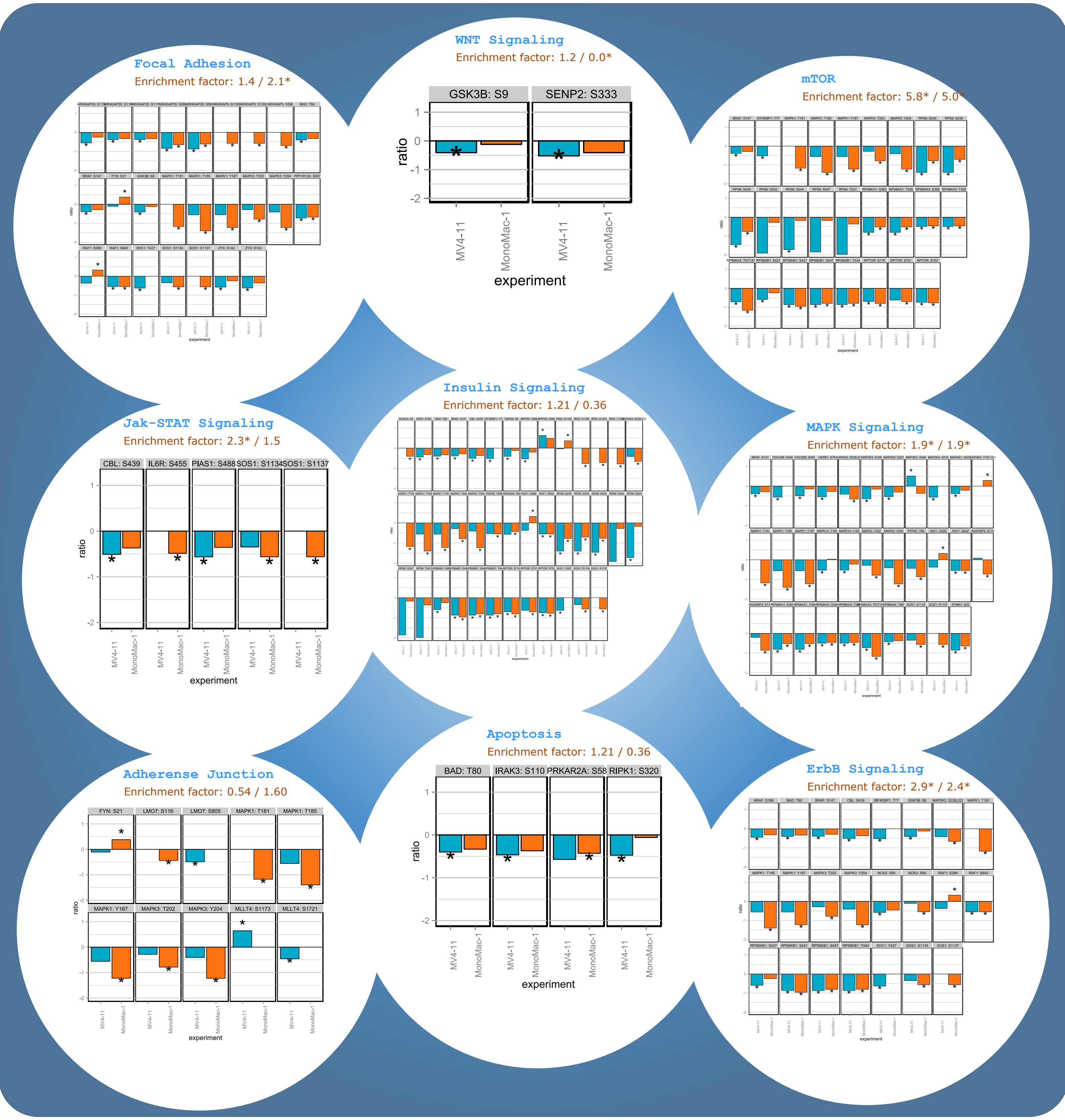

Focal Adhesion
Enrichment factor: 1.4 / 2.1*
WNT Signaling
Enrichment factor: 1.2 / 0.0*
GSK3B: S9
SENP2: S333
ratio
MV4-11
MonoMac-1
experiment
mTOR
Enrichment factor: 5.8* / 5.0*
Insulin Signaling
Enrichment factor: 1.21 / 0.36
Jak-STAT Signaling
Enrichment factor: 2.3* / 1.5
CBL: S439
IL6R: S455
PIAS1: S488
SOS1: S1134
SOS1: S1137
MAPK Signaling
Enrichment factor: 1.9* / 1.9*
Apoptosis
Enrichment factor: 1.21 / 0.36
BAD: T80
IRAK3: S110
PRKAR2A: S58
RIPK1: S320
Adherense Junction
Enrichment factor: 0.54 / 1.60
FYN: S21
LMO7: S116
LMO7: S805
MAPK1: T181
MAPK1: T185
MAPK1: Y187
MAPK3: T202
MAPK3: Y204
MLLT4: S1173
MLLT4: S1721
ErbB Signaling
Enrichment factor: 2.9* / 2.4*
