# Supplementary figures and images for: Phosphoproteome Analysis Reveals Differential Mode of Action of Sorafenib in Wildtype and Mutated FLT3 Acute Myeloid Leukemia (AML) Cells
Source: Mol Cell Proteomics. 2017 Apr 27;16(7):1365–76. doi: 10.1074/mcp.M117.067462 (PMC5500767; doi:10.1074/mcp.M117.067462)

# Transcription Factors

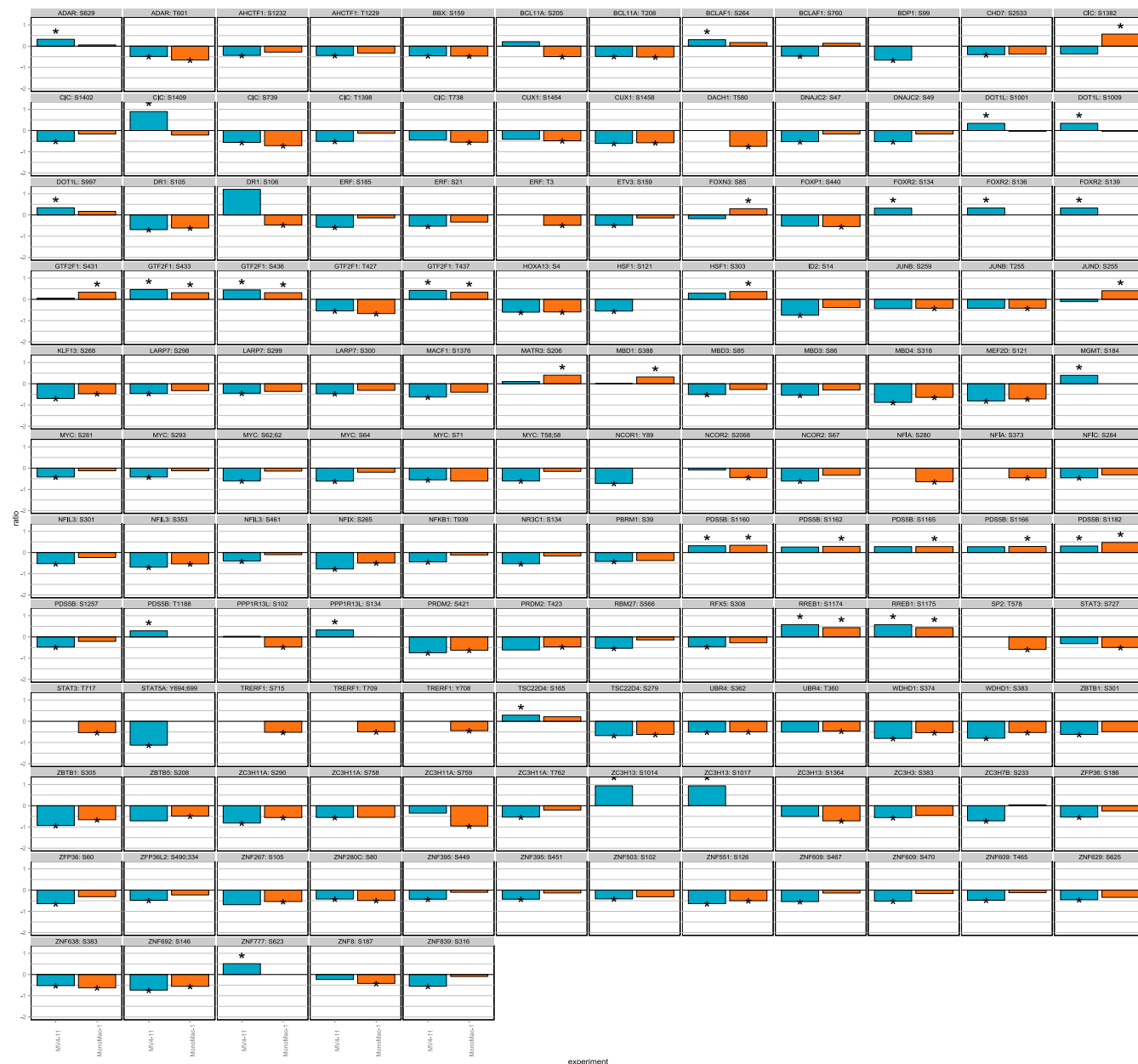

Supplement: Supplemental Data [file 10.1074_M117.067462_mcp.M117.067462-2.pdf]

# Translation Initiation Factors

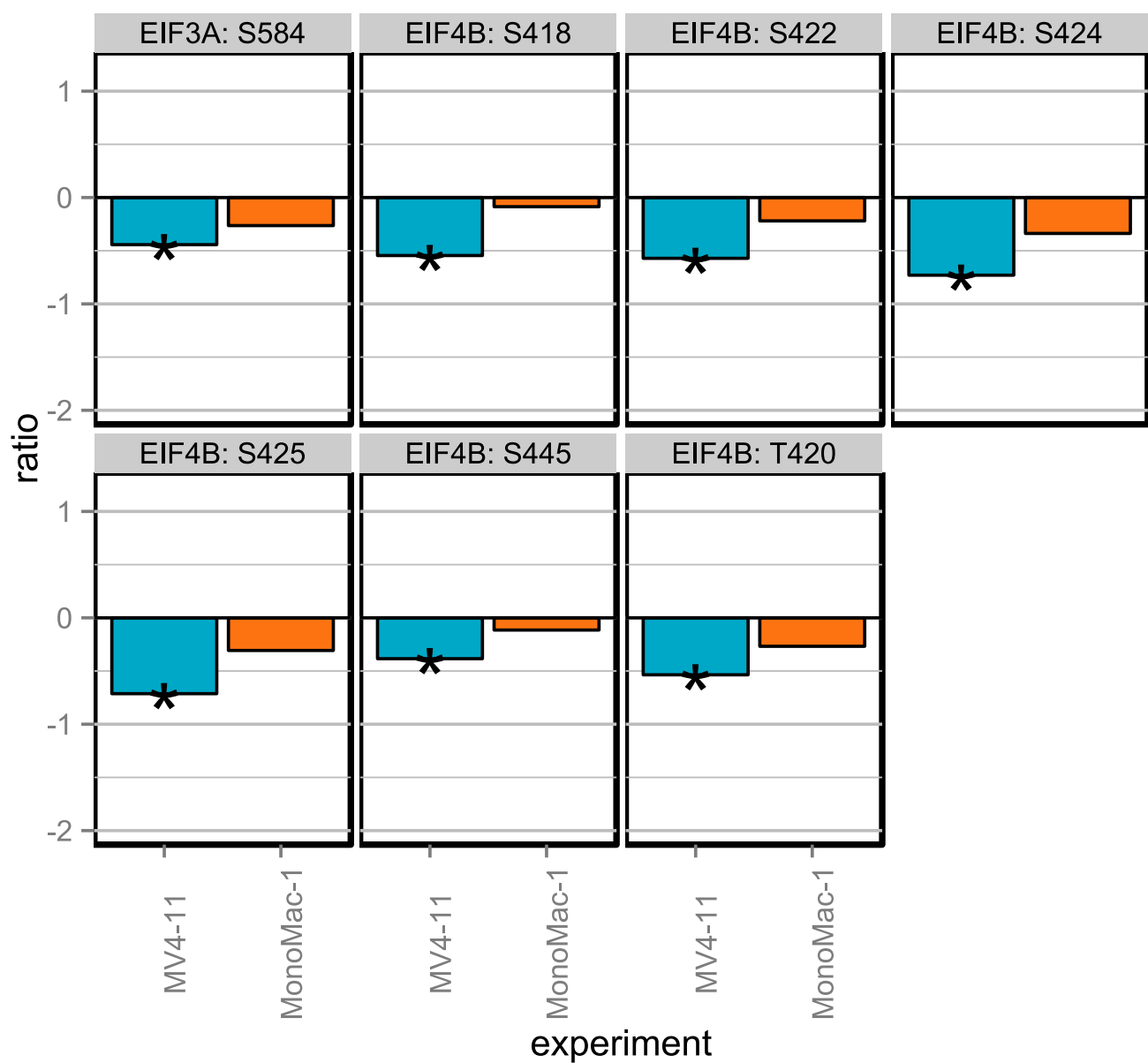

Supplement: Supplemental Data [file 10.1074_M117.067462_mcp.M117.067462-3.pdf]
